# AI-integrated multi-omics platform to revolutionize anti-metastatic therapy development through circulating tumor cell profiling: SCRUM-MONSTAR-CTC

**DOI:** 10.1007/s10147-026-03149-1

**Published:** 2026-07-27

**Authors:** Tadayoshi Hashimoto, Taro Shibuki, Takao Fujisawa, Shogen Boku, Mizuho Kikuchi, Atsuko Shimizu, Tomoe Murakami, Yasutoshi Sakamoto, Izumi Miki, Naoko Iida, Haruki Kurano, Riu Yamashita, Jun Masuda, Kensuke Matsuda, Junichiro Yuda, Shugo Yajima, Shin Kobayashi, Zedao Liu, Masataka Amisaki, Mitsuho Imai, Yoshiaki Nakamura, Hideaki Bando, Hyoju Kim, Heon Yung Gee, Hee Seung Lee, Hyun Woo Park, Takayuki Yoshino

**Affiliations:** 1 Translational Research Support Office, National Cancer Center Hospital East, Kashiwa, Japan; 2 Department of Gastrointestinal Oncology, National Cancer Center Hospital East, Kashiwa, 277-8577 Chiba Japan; 3 Perioperative Treatment Development Promotion Office, National Cancer Center Hospital East, Kashiwa, Japan; 4 Department of Hepato-biliary pancreatic Oncology, National Cancer Center Hospital East, Kashiwa, Japan; 5 Department of Head and Neck Medical Oncology, National Cancer Center Hospital East, Kashiwa, Japan; 6 Department of Clinical Oncology, Kansai Medical University Hospital, Hirakata, Japan; 7 Division of Translational Informatics, Exploratory Oncology Research & Clinical Trial Center, National Cancer Center, Kashiwa, Japan; 8 Breast Medical Oncology, The Cancer Institute Hospital of Japanese Foundation for Cancer Research, Tokyo, Japan; 9 Department of Hematology, National Cancer Center Hospital East, Kashiwa, Japan; 10 Department of Urology, National Cancer Center Hospital East, Kashiwa, Japan; 11 Department of Hepatobiliary and Pancreatic Surgery, National Cancer Center Hospital East, Kashiwa, Japan; 12 Department of Oncology, University of Oxford, Oxford, UK; 13 Department of Biochemistry, College of Life Science and Biotechnology, Brain Korea 21 Four Project, Yonsei University, Seoul, Republic of Korea; 14 Department of Pharmacology, Graduate School of Medical Science, Brain Korea 21 Project, Yonsei University college of medicine, Brain Korea 21 Project, Seoul, Republic of Korea; 15 Division of Gastroenterology, Department of Internal Medicine, Yonsei University College of Medicine, Seoul, Korea; 16 Global Center of Research Excellence for Advanced Medicine (G-CORE), Keio University School of Medicine, Tokyo, Japan

**Keywords:** Liquid biopsy, Circulating tumor cells, Metabolites, Anti-metastatic therapy, Precision Oncology, Metastatic plasticity

## Abstract

**Background:**

Metastatic disease remains the leading cause of cancer-related death, yet most precision oncology strategies still emphasize profiling primary tumors and tracking cell-free tumor DNA (ctDNA). Although ctDNA has transformed genomic profiling, molecular residual disease monitoring, and early cancer detection, it cannot directly capture viable tumor cell states, phenotypic plasticity, or functional adaptations that drive metastatic spread. We propose that the next phase of precision oncology should integrate the cellular dimension of metastasis through systematic circulating tumor cell (CTC) profiling.

**Methods:**

The SCRUM-MONSTAR platform, one of the largest pan-cancer molecular profiling initiatives in Japan, offers an exceptional foundation for this transition through its nationwide infrastructure for multi-omics analysis, longitudinal biospecimen collection, and artificial intelligence-enabled clinical interpretation. By combining matched tissue profiling, serial ctDNA analysis, single-cell CTC transcriptomics, metabolomics, and organoid- and mouse-based functional modeling, SCRUM-MONSTAR-CTC could evolve into a translational ecosystem for anti-metastatic drug discovery. Within this framework, we highlight adherent-to-suspension transition (AST) as one representative, experimentally tractable plasticity program that enables tumor cells to survive in circulation and subsequently colonize distant organs.

**Discussion:**

We envision that identifying and therapeutically targeting AST-related and other metastatic plasticity programs across tumor types will provide a path toward clinically actionable anti-metastatic therapies. More broadly, this framework could enable the identification of metastatic vulnerabilities, the development of biomarker-guided anti-metastatic trials, and the reverse translation of patient-derived discoveries into early-phase clinical testing. Precision oncology must move beyond cataloging tumor genomes and begin targeting metastasis as a dynamic biological process.

**Trial Registration:**

UMIN000056873, approved by the Institutional Review Board of the National Cancer Center Hospital East.

## Introduction

Metastatic disease accounts for most cancer-related deaths, yet our therapeutic logic remains disproportionately focused on the biology of primary tumors [1, 2]. This mismatch represents one of the most important unresolved problems in oncology. Targeted therapies and immunotherapies have improved outcomes in selected molecular subgroups, but metastatic progression continues to outpace therapeutic innovation because the mechanisms that enable dissemination, survival in circulation, dormancy, and distant colonization remain insufficiently characterized in patients.

The precision oncology era has been driven by tissue genotyping and, more recently, by liquid biopsy [3–5]. ctDNA has expanded our ability to detect actionable genomic alterations, monitor molecular residual disease, and identify recurrence earlier than imaging [6, 7]. These are major advances. However, metastasis is not governed by DNA sequence alone. It is a dynamic cellular process shaped by transcriptional plasticity, epithelial-to-mesenchymal and hybrid-state transitions, resistance to anoikis, immune evasion, and metabolic adaptation [8]. These features are poorly captured by ctDNA, which reflects fragmented nucleic acids shed from tumors rather than viable metastatic cells.

To close this gap, precision oncology must evolve from a largely genomics-centered model to a multimodal framework that captures genomic, cellular, spatial, and metabolic dimensions of disease. The SCRUM-MONSTAR platform provides an ideal foundation for this transition [9–11]. Built as a large-scale pan-cancer molecular profiling initiative in Japan, SCRUM-MONSTAR has already established the clinical infrastructure, biospecimen workflow, collaborative network, and data integration framework needed to support next-generation translational research at scale. We propose that its next strategic expansion should be the systematic incorporation of circulating tumor cell (CTC) analysis and functional modeling to create a platform for anti-metastatic drug discovery and clinical translation.

## SCRUM-MONSTAR as a foundation for metastasis-focused precision oncology

SCRUM-MONSTAR represents more than a profiling program. Its importance lies in its ability to connect nationwide clinical enrollment, comprehensive molecular analyses, and real-world clinical decision making [11]. The platform has progressively expanded from organ-focused screening to a broad pan-cancer ecosystem that integrates tissue sequencing, blood-based assays, clinicopathologic data, and emerging technologies. This architecture makes it uniquely suited to address metastasis, a problem that inherently requires longitudinal and multimodal observation.

Current SCRUM-MONSTAR activities already illustrate this trajectory. Tissue-based profiling can encompass whole-exome and whole-genome sequencing, transcriptome analysis, methylation profiling, and advanced spatial approaches. Blood-based initiatives are expanding the role of ctDNA for molecular residual disease assessment and early detection. The platform is also positioned to integrate imaging, electronic patient-reported outcomes, wearable data, and AI-supported clinical interpretation. Together, these components create a scalable precision oncology infrastructure. Yet one dimension remains underrepresented: the biology of the metastatic cell itself.

Tissue biopsies provide static snapshots from selected sites and time points. ctDNA enables dynamic genomic surveillance, but it does not reveal whether disseminating cells are epithelial, mesenchymal, stem-like, proliferative, dormant, immune evasive, or metabolically rewired. In other words, current precision oncology workflows detect traces of tumor-derived DNA, but they do not fully interrogate the cells actively executing the metastatic program. This is where CTC analysis becomes essential.

### Implementation of CTC profiling into SCRUM-MONSTAR

CTCs are not simply another liquid biopsy analyte. They are viable tumor cells that have entered the circulation and are therefore direct participants in the metastatic cascade [12, 13]. As such, they offer a rare opportunity to study the biology of dissemination in real time. Unlike ctDNA, CTCs can be interrogated at the single-cell level, enabling direct characterization of cellular heterogeneity, lineage plasticity, stress responses, and treatment-adaptive states [14].

This distinction is critical. The metastatic phenotype is shaped by reversible and non-genetic programs as much as by stable genomic lesions. CTCs can reveal epithelial-to-mesenchymal and hybrid phenotypes, cytoskeletal remodeling, suspension survival programs, stemness features, and surface protein expression patterns that may determine organ tropism or therapeutic sensitivity. These are precisely the features that remain largely invisible in ctDNA-based assays.

In addition, CTCs enable functional analysis. They can be expanded in ex vivo systems, used to generate organoids, and studied in mouse models [15, 16]. This creates a route from observation to mechanism and from mechanism to therapeutic testing. A metastatic platform built around CTCs could therefore achieve something that many liquid-biopsy studies cannot: a true translational loop linking biomarker discovery, biological validation, and drug development.

Metastasis is a multifactorial process governed by numerous, frequently overlapping programs, including epithelial–mesenchymal transition (EMT) and partial-EMT/hybrid states, collective cell migration, tumor dormancy, immune editing, and metabolic plasticity, each supported by substantial experimental and clinical evidence. Within this broader landscape, recent work highlights one representative, experimentally tractable program that is especially amenable to CTC-based study. A collaborative institution with SCRUM-MONSTAR described an adherent-to-suspension transition (AST) program in which defined hematopoietic transcriptional regulators reprogram anchorage dependency and enable solid tumor cells to disseminate as CTCs [17]. Mechanistically, AST suppresses integrin/extracellular matrix interactions through inhibition of the Hippo-YAP/TEAD axis and promotes resistance to oxidative stress and anoikis, thereby supporting blood-borne dissemination. Importantly, pharmacologic blockade of AST-related programs reduced CTC formation and distant metastasis without materially suppressing primary tumor growth. This is a critical conceptual advance: the relevant target may not be tumor bulk, but the biological state that permits dissemination. We emphasize, however, that AST is presented not as a unifying paradigm of metastasis but as one of several complementary mechanisms; a central strength of a CTC-informed platform is precisely its capacity to interrogate this spectrum of programs in parallel rather than privileging any single one. A national platform capable of interrogating CTC states longitudinally could therefore identify tractable vulnerabilities specific to metastasis rather than merely to proliferating primary tumors.

These observations also challenge the current biomarker paradigm. Conventional precision oncology matches therapies to genomic alterations detected in tissue or plasma. In metastatic disease, however, clinical behavior may be equally influenced by non-genomic states such as anchorage reprogramming, suspension survival, epithelial-mesenchymal or hybrid phenotypes, and immune-adaptive plasticity. A CTC-informed framework would therefore enable patient stratification not only by mutation, but also by dissemination phenotype.

## MONSTAR-CTC as an ancillary study in MONSTAR-SCREEN-3

In 2024, the SCRUM-MONSTAR consortium launched MONSTAR-SCREEN-3, a comprehensive molecular profiling initiative that integrates multi-modal approaches to characterize longitudinal and spatiotemporal molecular landscapes in malignant tumors. In this context, CTCs provide distinctive advantages through their capacity for comprehensive characterization at the single-cell level. To leverage these advantages, we designed MONSTAR-CTC as an ancillary within the MONSTAR-SCREEN-3 framework to specifically investigate the molecular architecture of CTCs and elucidate their potential clinical implications in advanced solid tumors. MONSTAR-CTC (UMIN000056873) will prospectively enroll 100 patients with advanced solid tumors during a 2-year accrual period, with subsequent follow-up for 1 year.

Enrollment began in February 2025, and completion of the planned 2-year accrual is anticipated by February 2027, followed by 1 year of follow-up. As of February 2026, 70 patients have been enrolled, supporting the feasibility of the target sample size. In our preliminary experience using the SMART BIOPSY™ platform, CTCs (DAPI⁺/EpCAM⁺/CD45⁻) were identified in 77.8% (21/27) of processed samples from the first 20 patients (27 samples in total: 20 collected before treatment and 7 at disease progression), with a mean of 10.3 CTCs per 5 mL of peripheral blood (range, 0–27). To ensure analytical quality, all procedures follow standardized operating procedures across participating institutions, including: (i) fixed pre-analytical handling, with blood processed within 6 h of collection; (ii) positive- and negative-control staining in each batch; (iii) independent image review for CTC enumeration; and (iv) pre-specified quality thresholds for single-cell libraries (minimum captured genes per cell, mitochondrial-read fraction, and library complexity), with cells or samples failing these thresholds excluded from downstream analysis. Samples not meeting the minimum CTC yield will be systematically recorded to allow transparent reporting of the overall analytical success rate.

**Fig. 1:**
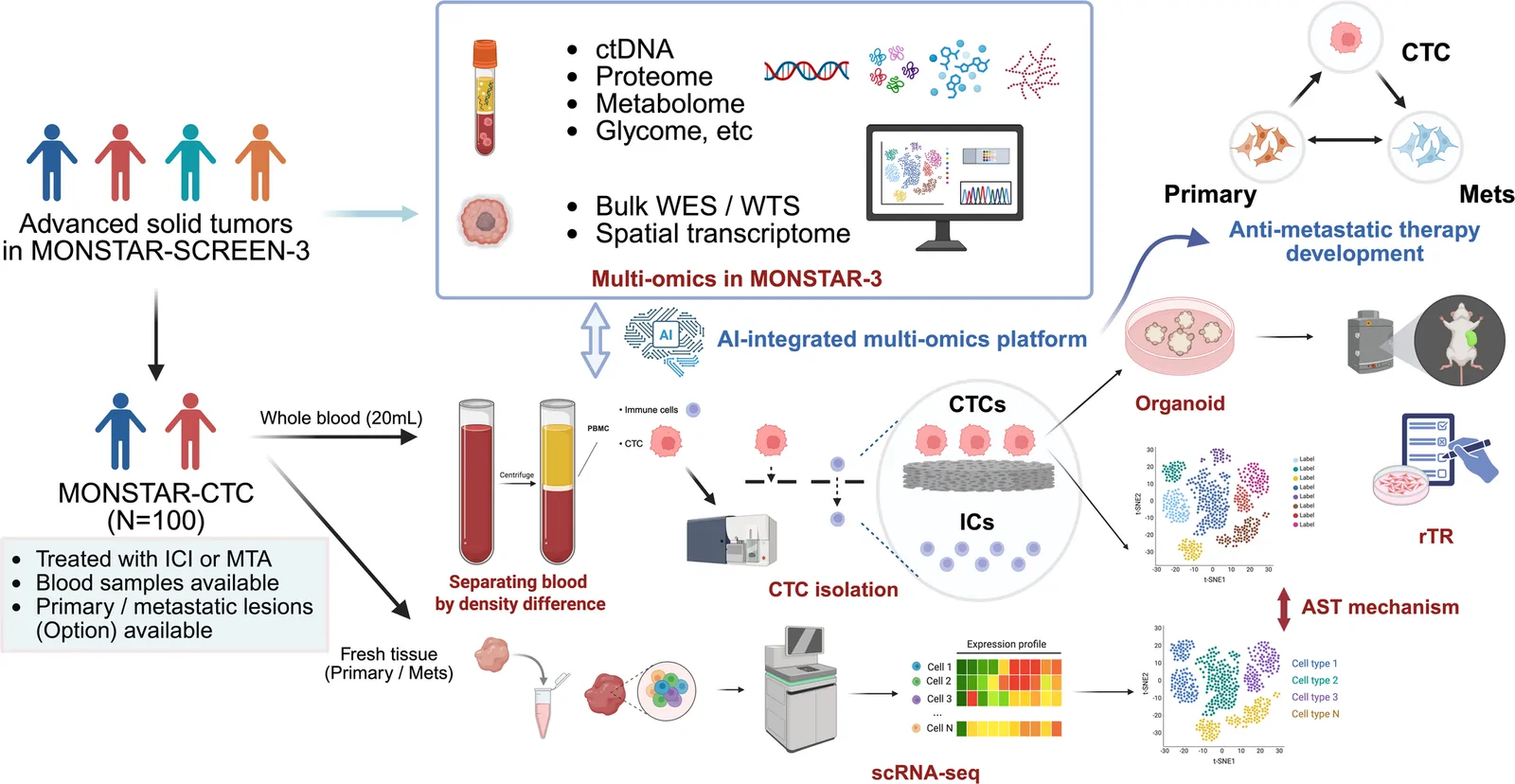
SCRUM-MONSTAR-CTC framework. MONSTAR-CTC is an ancillary study within the MONSTAR-SCREEN-3 framework designed to investigate the molecular architecture of circulating tumor cells (CTCs) and their clinical implications in advanced solid tumors. The study will prospectively enroll 100 patients with advanced solid tumors (UMIN000056873). Blood samples are processed for CTC enrichment using a CTC isolation kit and SMART BIOPSY™ Cell Isolator. Multi-omics analyses — including whole exome/transcriptome sequencing, spatial transcriptomics, and single-cell transcriptomics — are performed on matched specimens from primary lesions, CTCs, and metastatic lesions. This integrated framework aims to advance precision oncology beyond primary tumor-based approaches toward more effective strategies for patients with metastatic disease. Matched organoid and mouse models derived from CTCs and tumor tissue support mechanistic studies and functional testing of anti-metastatic therapeutic hypotheses. AI-driven multimodal integration links these data layers to identify biomarker-defined metastatic states, prioritize drug targets, and guide biomarker-driven early-phase trials

The study implements multi-omics analyses in matched primary lesion, CTCs, and metastatic lesions (Optional) comprising whole exome/transcriptome sequencing, spatial transcriptomics, and single-cell transcriptomics of tissue specimens. Peripheral blood samples were collected and processed for CTC enrichment using a CTC isolation kit (Cytogen, CIKW10) in combination with the SMART BIOPSY™ Cell Isolator (Cytogen, CIS030) [17–19]. Enriched cells were cytospun onto glass slides using a Cytospin 4 cytocentrifuge (Thermo Fisher Scientific) and subjected to multi-immunofluorescence staining for CTC identification. Nucleated cells were identified by staining with 4′,6-diamidino-2-phenylindole (DAPI), while leukocytes were detected using an anti-CD45 antibody. CTCs were defined as DAPI-positive, epithelial cell adhesion molecule (EpCAM)-positive, and CD45-negative cells. Fluorescence images were acquired and analyzed using the SMART BIOPSY™ Cell Image Analyzer (Cytogen, CIA030) and its accompanying image analysis software. When clinically feasible, matched tissue specimens from both primary and metastatic sites will enable triangulated molecular analyses across different tumor compartments. The primary objective is to evaluate associations between CTC molecular signatures and clinical outcomes, including treatment response, minimal residual disease, and recurrence. Secondary objectives include: (1) exploration of correlations between CTC molecular characteristics and multi-omics data; (2) elucidation of fundamental metastasis mechanisms; (3) identification of novel therapeutic targets for metastasis treatment; and (4) development of integrated liquid biopsy approaches combining CTC and circulating tumor DNA analyses that predicts metastasis. This integrated multi-modal approach has the potential to identify novel biomarkers for treatment selection and metastasis mechanisms, ultimately advancing current primary tumor-based precision oncology to develop more effective therapeutic strategies for patients with metastatic disease (Fig. 1).

## Perspective of SCRUM-MONSTAR-CTC framework

We envision a dedicated expansion of the platform, termed SCRUM-MONSTAR-CTC, embedded within the broader SCRUM-MONSTAR ecosystem. This effort would not replace existing tissue or ctDNA programs; rather, it would complement them by adding the cellular layer of metastatic biology.

The first step is standardized longitudinal blood collection, ideally at baseline, during treatment, at major response assessment time points, and at progression or recurrence. CTC enrichment should use approaches broad enough to capture epithelial as well as mesenchymal or hybrid populations, since exclusive reliance on epithelial markers risks overlooking the very cells most relevant to metastasis. These enriched cells could then undergo single-cell transcriptomic analysis and, where feasible, combined RNA and surface protein profiling.

Beyond transcriptomic characterization, single-cell surface-protein and proteomic profiling of CTCs may directly inform antibody-based therapeutic strategies. Because CTCs are the viable cells actively disseminating, their surface-antigen repertoire (e.g., HER2, TROP2, CEACAM5, and other actionable targets) may identify candidates for antibody–drug conjugates (ADCs) and related modalities, and may capture target expression that is discordant with the primary tumor. This approach could be especially valuable in the adjuvant and molecular-residual-disease settings, where biomarker-guided selection of patients harboring disseminated, ADC-targetable cells could enable rational, minimally invasive treatment stratification before overt metastatic relapse.

The true power of this framework emerges when CTC data are analyzed alongside matched tissue and ctDNA. Primary tumors define the originating genomic and spatial context. ctDNA captures longitudinal tumor-derived genomic change across disease sites. CTCs identify the cellular phenotypes actively disseminating or adapting under treatment pressure. When integrated, these modalities can reveal which genomic alterations associate with specific metastatic cell states, which cellular programs emerge despite stable genomics, and which vulnerabilities are retained as tumors evolve.

Metabolism should be incorporated as a fourth major pillar [20]. Metastatic competence depends not only on signaling and plasticity but also on bioenergetic fitness. CTC survival in circulation, extravasation, and colonization likely require metabolic rewiring that differs from primary tumor growth. Integrating plasma metabolomics with CTC single-cell data could reveal systemic metabolic signatures linked to dissemination, dormancy, or therapy resistance. Such profiling may identify actionable dependencies, including nutrient scavenging pathways, oxidative stress tolerance, and lipid or amino acid requirements, thereby opening a path to anti-metastatic metabolic therapies.

The power of this design lies in integration [21]. Tissue profiling defines the originating genomic and spatial context. ctDNA captures longitudinal tumor-derived genomic evolution across disease sites. CTCs identify viable dissemination-competent cell states. Metabolomics adds the biochemical context that may support or constrain those states. In this context, we outline a concrete analytical strategy using artificial intelligence (AI) that combines complementary learning paradigms. Unsupervised and self-supervised methods, including dimensionality reduction, clustering, and self-supervised single-cell representation learning, will be used to define CTC cell states and to derive embeddings without reliance on predefined labels, which is essential given the exploratory nature of dissemination phenotypes. Supervised models will subsequently link these representations to clinical endpoints such as treatment response and recurrence. For cross-modal integration of matched tissue, ctDNA, single-cell CTC, and metabolomic data, we are evaluating multimodal transformer-based architectures and graph neural networks, the latter to model cell-cell and feature-feature relationships, alongside emerging single-cell foundation models for transfer learning as the atlas scales. All models will undergo cross-validation and, where feasible, external validation, with particular attention to interpretability and to mitigating overfitting in the setting of high-dimensional, modest-sample data. AI-driven analysis can then connect these layers to derive composite biomarkers of metastatic risk, therapeutic resistance, and anti-metastatic vulnerability. This is the natural next step for a platform such as SCRUM-MONSTAR: not merely accumulating more data but integrating orthogonal dimensions of disease biology into a clinically actionable model.

## Translational and reverse-translational opportunities

The greatest value of MONSTAR-CTC would be its ability to support an end-to-end translational ecosystem. Patient-derived CTCs and matched tissue samples could be used to generate organoids and mouse models that recapitulate metastatic behavior more faithfully than conventional primary-tumor systems. These models would enable mechanistic study of dissemination, colonization, and therapeutic escape. More importantly, they would provide experimental platforms for anti-metastatic drug discovery.

This would establish a translational–reverse translational pipeline in which clinical samples generate biological models, those models uncover therapeutic vulnerabilities and resulting candidate drugs or combinations are then brought back into biomarker-guided early-phase trials. Such a framework is particularly well suited to anti-metastatic therapy development, where conventional drug discovery has been limited by the lack of clinically relevant models of dissemination and colonization.

This ecosystem could support several classes of emerging therapeutics: agents targeting CTC-enriched surface antigens; drugs that disrupt epithelial-mesenchymal plasticity or suspension survival programs; metabolic inhibitors aimed at dissemination-specific dependencies; and immunologic strategies designed to enhance clearance of disseminated tumor cells. By linking these approaches to multimodal biomarkers, SCRUM-MONSTAR could provide the patient-selection and pharmacodynamic framework needed to test anti-metastatic therapies rationally rather than empirically.

At present, no CTC-guided clinical trial is being conducted within the SCRUM-MONSTAR-CTC framework; MONSTAR-CTC is designed primarily to establish the biological and technical foundation required to enable such trials. Building on this foundation, we anticipate two complementary directions. First, systematic single-cell CTC profiling could enable the design of CTC-driven, biomarker-guided trials, in which patients are selected or stratified according to dissemination-relevant cell states and surface-antigen expression rather than by tissue genotype alone. Second, the platform is intended to support the clinical translation of anti-metastatic agents currently under development, including inhibitors targeting the AST and related plasticity programs. We are planning early-phase studies to evaluate the safety and preliminary efficacy of such anti-metastatic candidates, using platform-derived CTC and multi-omics readouts as pharmacodynamic and patient-selection biomarkers. In this way, the SCRUM-MONSTAR-CTC platform is positioned to move candidate anti-metastatic strategies from biological discovery toward rationally designed, biomarker-guided clinical testing.

## Implications for clinical trial design

The success of this strategy will depend on standardization, collaboration, and regulatory alignment. Sample handling, CTC isolation, single-cell analysis, and metabolomic workflows must be reproducible across institutions. Biomarker development will require rigorous analytical validation and prospective clinical qualification. Industry partnerships will be essential to connect discovery with drug development, especially for therapies directed at dissemination-specific surface markers, cell-state regulators, or metabolic dependencies. Yet these challenges are precisely the kind that national precision oncology infrastructures are designed to address [21].

The broader significance of this vision extends beyond one platform. SCRUM-MONSTAR offers a blueprint for how large clinically embedded multi-omics networks can evolve from profiling programs into therapeutic discovery engines. The field has already learned that ctDNA can transform non-invasive cancer monitoring. The next leap may come from combining ctDNA with direct analysis of viable metastatic cells and their metabolic environment. Doing so would align precision oncology more closely with the biologic process that causes most cancer deaths.

### Perspective and Conclusion

Precision oncology has transformed cancer care, but it still does not adequately address the biology that kills most patients: metastasis. ctDNA has opened an essential new era of blood-based oncology, yet it cannot by itself capture the viable cellular states that drive dissemination, colonization, and treatment escape. The next major advance will come from integrating genomics with direct profiling of metastatic cells.

SCRUM-MONSTAR is exceptionally well positioned to lead this transition. By incorporating CTC single-cell analysis, metabolomic profiling, organoid and mouse modeling, and AI-driven multimodal integration, the platform could evolve from a national molecular profiling initiative into a comprehensive anti-metastatic drug development engine. Such an expansion would not only deepen our biological understanding of metastatic disease but also create a practical route to biomarker-guided early-phase trials and, ultimately, a new class of therapies designed to target metastasis itself.

In this sense, the future of precision oncology is not simply to profile more mutations. It is to understand, intercept, and therapeutically exploit the dynamic cellular process of metastasis. SCRUM-MONSTAR can help make that future real.

## Data Availability

To protect patient privacy and confidentiality, the clinical and activity tracking data from this study are not publicly available. However, de-identified data may be available from the corresponding author upon reasonable request and with appropriate approval from the study steering committee. Any data sharing requests will be reviewed to verify whether the request is subject to any intellectual property or confidentiality obligations. Figures were created with BioRender software (HASHIMOTO, T. (2026) https://BioRender.com/w8n2z7x).

## Abbreviations

AI: Artificial intelligence
AST: Adherent to suspension transition
CTC: Circulating tumor cell
ctDNA: Circulating tumor DNA
IC: Immune cell
ICI: Immune-checkpoint inhibitor
Mets: Metastatic sites
rTR: Reverse translational research
PBMC: Peripheral blood mononuclear cells
scRNA-seq: Single-cell RNA sequencing
MTA: Molecular-target agent
WES: Whole exome sequencing
WTS: Whole transcriptome sequencing
